# Supplementary material for: Protective efficacy of holed and aging PBO-pyrethroid synergist-treated nets on malaria infection prevalence in north-western Tanzania
Source: PLOS Glob Public Health. 2022 Oct 17;2(10):e0000453. doi: 10.1371/journal.pgph.0000453 (PMC10022078; doi:10.1371/journal.pgph.0000453)
Supplement: S1 Table — (DOCX) [file pgph.0000453.s001.docx]

S1 Table. Survivorship and attrition of cohort nets by age and arms with and without Indoor Residual Spraying (IRS) (data from longitudinal survey)

 Attrition rate-category1: for nets that have been destroyed or disposed of due to wear and tear (poor condition) in surveyed households; Attrition rate-category2: for nets not available for sleeping under for reasons other than poor fabric integrity (given away, stolen, sold or used in another location, withdrawn by PAMVERC staff) in surveyed households; Attrition rate-category3: for nets used for other purposes in surveyed households; * Missing nets from households that were not interviewed due to either (dwelling vacant, a dwelling not found, and refused) and were not included in the denominator when calculating survivorship and attrition
