# Supplementary material for: Protective efficacy of holed and aging PBO-pyrethroid synergist-treated nets on malaria infection prevalence in north-western Tanzania
Source: PLOS Glob Public Health. 2022 Oct 17;2(10):e0000453. doi: 10.1371/journal.pgph.0000453 (PMC10022078; doi:10.1371/journal.pgph.0000453)
Supplement: S2 Table — A. Proportion of net still present in the house and in serviceable condition (= functional survival) by net product and time point and median survival in years. B. Cox regression analysis presenting unadjusted and adjusted Hazard ratio. (DOCX) [file pgph.0000453.s002.docx]

S2A Table. Proportion of net still present in the house and in serviceable condition (=functional survival) by net product and time point and median survival in years

| **Net product** | **Percent functional survival (95% CI), N** | | | **Median survival in years (95% CI)** |
| --- | --- | --- | --- | --- |
|  | **12 months** | **24 months** | **36 months** |  |
| Standard LLIN (Olyset net) | 88.0 (85.4-90.1), N=329 | 42.4 (35.9-49.3), N=308 | 17.9 (14.7-21.5), N=309 | 1.85 (1.67-2.06), N=348 |
| PBO LLIN (Olyset plus) | 86.7 (65.2-95.8), N=326 | 37.2 (18.5-60.7), N=313 | 15.4 (4.8-39.7), N=309 | 1.63 (1.38-1.87), N=368 |

p-Value for the comparison between 2 nets functionally surviving, p=0.7971, p=0.5371, and p=0.6929 at 12, 24 and 36 months, respectively

S2B Table. Cox regression analysis presenting unadjusted and adjusted Hazard ratio

| Net product | Total net | Number of event = net lost | Unadjusted Hazard ratio (95% CI), p-value | *Adjusted Hazard ratio (95% CI), p-value |
| --- | --- | --- | --- | --- |
| Standard LLIN (Olyset net) | 348 | 276 (79.3%) | 1.0 | 1.0 |
| PBO LLIN (Olyset plus) | 368 | 301 (81.8%) | 1.13 (0.86-1.48), p=0.39 | 1.14 (0.96-1.35), p=0.15 |

*adjusted for IRS (with or without IRS)
