# Supplementary material for: Protective efficacy of holed and aging PBO-pyrethroid synergist-treated nets on malaria infection prevalence in north-western Tanzania
Source: PLOS Glob Public Health. 2022 Oct 17;2(10):e0000453. doi: 10.1371/journal.pgph.0000453 (PMC10022078; doi:10.1371/journal.pgph.0000453)
Supplement: S3 Table — (DOCX) [file pgph.0000453.s003.docx]

S3 Table. Mean (geometric) number of holes by zone (location) and by size

NB: Standard LLIN (Olyset Net), PBO LLIN (Olyset Plus)
