# Supplementary material for: Protective efficacy of holed and aging PBO-pyrethroid synergist-treated nets on malaria infection prevalence in north-western Tanzania
Source: PLOS Glob Public Health. 2022 Oct 17;2(10):e0000453. doi: 10.1371/journal.pgph.0000453 (PMC10022078; doi:10.1371/journal.pgph.0000453)
Supplement: S4 Table — (DOCX) [file pgph.0000453.s004.docx]

S4 Table: Median survival of the nets by study arms

| **Net product** | **Median survival in years (95% CI), N** | **p-values for Log-rank test for equality of survivor functions** |  |
| --- | --- | --- | --- |
|  |  |  |  |
| **Comparison between net arms regardless of IRS status** | | |  |
| Standard LLIN arms (Olyset net) | 1.85 (1.67-2.06), N=348 | p=0.1487 |  |
| PBO LLIN arms (Olyset plus) | 1.63 (1.38-1.87), N=368 |  |  |
| **Comparison between IRS arms regardless of net type** | | |  |
| Non-IRS arms | 1.89 (1.83-1.99), N=356 | p=0.0103 |  |
| IRS arms | 1.62 (1.38-1.83), N=360 |  |  |
| **Comparison between the four arms** |  |  |  |
| Standard LLIN (Olyset net) without IRS | 1.89 (1.67-2.05), N=179 | p=0.0036 |  |
| Standard LLIN (Olyset net) with IRS | 1.83 (1.40-2.08), N=169 |  |  |
| PBO LLIN (Olyset plus) without IRS | 1.97 (1.65-2.07), N=177 |  |  |
| PBO LLIN (Olyset plus) with IRS | 1.40 (1.21-1.63), N=191 |  |  |
