# Supplementary material for: Protective efficacy of holed and aging PBO-pyrethroid synergist-treated nets on malaria infection prevalence in north-western Tanzania
Source: PLOS Glob Public Health. 2022 Oct 17;2(10):e0000453. doi: 10.1371/journal.pgph.0000453 (PMC10022078; doi:10.1371/journal.pgph.0000453)
Supplement: Attachment — Submitted filename: Response to Reviewers_Eliud Lukole.docx [file pgph.0000453.s006.docx]

Dear Editor,

We thank the reviewers for the thorough reviews. Please find below our point by point revision of the manuscript (PGPH-D-22-00608) entitled “Protective efficacy of holed and aging PBO-pyrethroid synergist-treated nets on malaria infection prevalence in north-western Tanzania”

Journal Requirements:

1. Thank you for indicating that the study reported within the manuscript text is a secondary analysis of the clinical trial registered: NCT02288637. For the purposed of transparency please clearly indicate this within the manuscript text. Furthermore it is also not necessary to provide the study protocol as supporting information, we recommend that this document is removed.

**We have removed the study protocol and clearly stated in the manuscript at the end of the Introduction**

2 .Please amend your detailed Financial Disclosure statement. This is published with the article. It must therefore be completed in full sentences and contain the exact wording you wish to be published.

State the initials, alongside each funding source, of each author to receive each grant.

**Done**

3. Please update your online Competing Interests statement. If you have no competing interests to declare, please state: “The authors have declared that no competing interests exist.”

**Done**

4. In the online submission form, you indicated that “Deidentified data will be made available upon reasonable request to the corresponding author. There is no legal or ethical restrictions to the data.”. All PLOS journals now require all data underlying the findings described in their manuscript to be freely available to other researchers, either 1. In a public repository, 2. Within the manuscript itself, or 3. Uploaded as supplementary information.

This policy applies to all data except where public deposition would breach compliance with the protocol approved by your research ethics board. If your data cannot be made publicly available for ethical or legal reasons (e.g., public availability would compromise patient privacy), please explain your reasons by return email and your exemption request will be escalated to the editor for approval. Your exemption request will be handled independently and will not hold up the peer review process, but will need to be resolved should your manuscript be accepted for publication. One of the Editorial team will then be in touch if there are any issues.

**After enquiries we would like to modify our statement. Data in Tanzania are governed by strict law and cannot be share outside the country without a Data Transfer agreement (**[**DTA.pdf (nimr.or.tz)**](http://reims.nimr.or.tz:8010/guides/DTA.pdf)**) and appropriate ethical approval. There was no provision under this study to share the data widely outside and was not mentioned in the consent form. It might be difficult to share widely without a DTA process.**

5. Please provide separate figure files in .tif or .eps format and ensure that all files are under our size limit of 10MB.

For more information about how to convert your figure files please see our guidelines: https://journals.plos.org/globalpublichealth/s/figures

**Thank you for this. All figures are now in .tif format.**

Reviewer #1: This manuscript presents the analysis of a dataset generated on net longevity and effectiveness from a clinical trial conducted in Tanzania. The manuscript covers a generally well conducted and important study and provides important information for malaria vector control programs and researchers. Its valuable and presents important details to researchers, though some of the effectiveness information has been previously discussed in other reports. Nevertheless, this study presents significant added value to those earlier reports on the main trial findings.

There are a number of minor points and few major issues that should be addressed before this manuscript is fully ready for publication.

Major issues:

1) The authors present findings from net longevity in the context of a factorial RCT. The authors present findings on net longevity and also note that in two of the four trial arms, the ITNs were combined with IRS. They note that having IRS alongside of the ITNs significantly decreased net longevity. This finding is very important for the overall study and may be one of the more important findings of the entire paper as it has significant policy implications for the use of combination interventions, which the authors note. Despite this, It seems that they present unadjusted net longevity estimates, (at least I assume) averaging across the nets where IRS was present and where IRS was not present. While these are likely statistically accurate representations of the nets in the trial, doing so greatly reduces the generalizability of their findings. IRS is deployed in a relatively limited scope, is generally not recommended to be done in combination with ITNs by WHO, and therefore, the estimates of net lifetimes without IRS would be much more helpful for the general malaria community to make use of these findings (the authors could at least make the focus showing their adjusted results so that readers could see both).

**We thank the reviewer for this comment and agree that this is an important and potentially surprising result. As such, we ensured that in the final analysis we always adjusted for whether IRS was present or not. Looking only in the arm where nets were present without IRS will greatly reduce the sample size and therefore the precision of the estimates. However, we acknowledge that for practical reasons implementors may be more specifically interested in the net only estimates, so we have now included these in the supplementary data (S4 Table).**

2) The corollary to the above is that there appear to have been significant numbers of non-study nets available in the study area. The authors recognize that participants may have substituted IRS coverage for ITNs leading to a lower durability in the IRS arm. But they do not discuss or acknowledge that similar phenomenon may have occurred with availability of non-study nets as well. The availability of new non-study nets, might also affect lifetimes of nets in a similar way, leading to earlier disposal of less damaged nets when replacements are available.

**We thank the reviewer for making a useful point and we had tried to allude to this in our discussion, however we have now made it more explicit. A sentence has been added on page 26 line 349-352 “A similar effect may have been seen in clusters where non-study nets were readily available to replace lightly damaged study nets- the durability of the nets therefore may be underestimated in settings where other interventions are abundant.”**

3) Lastly there are a couple of statements in the conclusion that are not well founded in the evidence presented in the manuscript.

**The conclusion has been revised as it was suggested in below under CONCLUSION comments.**

4) Since one of the main outcomes of the study is median lifetime, it would be very useful to see the kaplan meir plots for lifetimes of nets, rather than only the plot of of the insecticide decay chart.

**We thank the reviewer for this useful suggestion. The Kaplan Meier plots for the life time of nets is now added as supplementary figure (S1 Figure)**

More minor concerns:

First paragraph of study design: This is mildly confusing, the heading is longitudinal LLIN study, but the paragraph describes two studies, a cohort study and a repeated cross sectional studies. I would suggest that the former is a longitiduinal study and the latter is a cross sectional study. The authors could do a better job making these two studies/data collection modalities stand out.

**We have now simplified the descriptions to make this clearer by separating out the two descriptions in this section on page 6 line 124-125.**

last line of this paragraph, nets chosen for sampling which had already been "attritted" were replaced with existing nets. This is fine as a study design, but it leads to an overestimate of how much insecticide and or intact fabric remains over time, because it is an estimate of how much insecticide and intact fabric are there on nets that remain in use in the original house. THe authors need to be careful to frame all these results in this context.

**We recognise that replacing the sampled net introduces newer nets in the community, however those new nets were not included in the net cohort follow up and therefore won’t have introduced a bias. We have changed the sentences in the paragraph to clarify on page 5-6 line 117-122 under Longitudinal LLIN study. The following sentence has been added to make this clearer:**

**“****In addition, 10 PBO LLIN and 10 standard LLINs still in use were randomly sampled from the households for insecticide content analysis and if the selected nets were missing, additional nets were sampled. All sampled nets were replaced with new nets of the same brand but were not subsequently followed up for fabric integrity or chemical analysis”**

Data analysis, svy command used to adjust SEs for clustering at RCT clusters. Were there more than one net per household in study? If so was SE inflation for within household clustering considered? IT seems that there would be significant within household correlation based on the association of SES and net lifetime alone.

**We thank the reviewer for this comment. Yes, there were more than one net per household, however, we have adjusted for the highest level of clustering (cluster), rather than the household. This is a standard way of doing the analyses.**

Functional survival over time was calculated using cox regression? Usually this is done with a KM estimator? COX regression would be used to estimate hazard ratios associated with determinants of hazard.

**KM was used to estimate the median lifetime of net while Cox estimate the hazard ratio for the difference in functional survival between nets**

The use of multivariate as a term for a regression generally implies multiple outcomes. IT is probably not the right term for the logistic regressions here multiple logistic or multivariable logistic regression is probably more accurate. Especially if this logistic regression is not specified using a variance components model, as seems to be the case here where the svy: prefix was used in STATA rather than a Random effects estimator.

**This has been corrected.**

Net physical integrity section: There is a error printout for a missing reference in the manuscript.

**This has been corrected and updated to Table 4, line 249.**

last sentence of this section, sentence should not start with however. This sentence is difficult to understand and should be re-written.

**The sentence has been revised on (page 18, line 271-272) and now it reads as “In the longitudinal data, net age, unfolding the net in the morning, and sleeping without a bed frame were significantly associated with net damage.”**

Chemical content of nets: Careful to frame these results in the context of this is only assessed among nets which remain in use and identifiable and as such is probably an overestimate of the remaining insecticide that was initially distributed. (i.e. this analysis has a survivor bias built in).

**We thank the reviewer for an interesting point however, we do not feel that this would be the case. Nets are usually discarded based on their physical integrity by the owner rather than suspecting that the insecticide contents has reduced. In addition, the opposite may also be true in that older nets are more likely to have been washed more, been exposed to the sun and daily use.**

First paragraph of discussion... missing references.

**Thank you for noticing this. The references have been added to the manuscript. Reference (18, 28) on line 314.**

IN general: the discussion contains some sloppy wording. The authors should be careful to use the term "MEDIAN Lifetime" or something specific which relates to their analysis rather than simply saying "the nets don't last three years" throughout. This lack of precision leads to divorcing their findings form their conclusions and a lack of clarity around how big this discrepancy actually is.

**We have now reviewed the discussion and hope we have clarified our meanings.**

First sentence of second discussion paragraph: This is very hard to follow and probably a run on sentence, should be rewritten.

**Thank you for noticing this. The sentence has been revised and rewritten on line 317-319, and now reads as “The majority of nets followed in the longitudinal study had at least one hole by 12 months with poor fabric integrity states as the main reason for attrition.”**

"examined by the field workers were actually still in serviceable condition. Community attitudes towards damaged nets may vary from place to place and may feed into differences in durability seen across countries(30)." - please say were in serviceable condition per WHO guidelines or something, they were not considered serviceable by the households.

**Thank you for noticing this. Here we were using the word serviceable as per the WHO guidelines. The sentence has been revised on page 25 line 328-331 and it now reads as “examined by the field workers were actually still determined as serviceable condition per WHO guidelines. Community attitudes towards damaged nets may vary from place to place and may feed into differences in durability reported between countries(28)”**

"PBO nets had less functional survival by 12 months, suggesting that users had held onto them even if they were in bad condition" – I found this sentence very hard to parse. Would suggest rephrasing.

**This has been revised and it is now on page 25-26 line 333-336 and reads “The higher survivorship (presence of nets in the household) despite fewer being classed as in serviceable condition per WHO guidelines of PBO nets by 12 months, suggests that users held onto them even if they were in bad condition”.**

"This is not a chance finding since communities were randomly allocated to receiving IRS, and may therefore have implications for any future strategy that combines different interventions." - RCTs can still result in findings by chance. But I agree that this is likely due to the effect of having IRS in those areas, that indeed it isn’t due to chance. It also reinforces the idea that substitute goods such as IRS or new nets might serve to reduce the lifetime of nets. The authors report this for IRS but neglect it as a threat to generalizability of findings....

**In response to the reviewer’s previous comments, we have now added a sentence about the issue of substitute goods perhaps leading to an under-estimation of the results. We have also included the results separated by arm in (S4 Table). We have reworded the original sentence to say “This is unlikely to be a chance finding since communities were randomly allocated to receiving IRS, and may therefore have implications for any future strategy that combines different interventions” (page 26, line 347-352)**

"Therefore, to maximize the gain that PBO nets offer in combating malaria, programmes and funding agencies should consider a two-year cycle for net replacement strategies. " - THIS Statement should go out, it does not follow scientifically or logically from their findings. The relative gains from deploying PBO nets compared to non-PBO nets of a similar age are indeed greater in the second year, but this tells us nothing about what cycle a campaign would follow to maximize an undefined "gains." Leaving aside that maximizing gains without some accompanying constraint such as a budget ceiling is not a sensible goal since it leads to the logical conclusion of a one year cycle or a three week cycle being even better than a two year cycle, the idea that using a two year cycle rather than a three or four or one year cycle would maximize gains, relies only on the relative gains of arm A vs. B here. e.g. a two year cycle of PBO distribution, compared to a two year cycle of standard net distribution would in this case create the maximum difference in gains between the two alternatives. This is not the same thing as maximum gains (no matter how you describe them).

**We have modified our statement however, we feel strongly that net replacement strategies should match the durability of the nets or that net durability should be improved to maximise impact even in the third year after net distribution if a 3 year cycles is proposed.**

**We have modified the sentence on (page 27 line 367-372) :**

**“****Therefore, to protect the increased efficacy that PBO nets offer in combating malaria, programmes and funding agencies should consider adapting net distribution strategies to the effective net lifespan or alternatively physical and chemical durability of the nets need to be improved to meet the three years replacement cycle. Currently, it seems clear that swathes of the community are using considerably less effective nets for at least a year before the next net distribution cycle (40) “**

"These findings concur with the earlier findings on torn PBO LLIN, that they will continue protecting against blood-feeding Anopheles irrespective of physical condition(31)." - It strains credulity that this is "Irrespective" of physical condition.... but agree that damaged nets could still protect. Suggest rewording.

**The sentence has been revised and now it reads “These findings concur with the earlier findings on torn PBO LLIN, that they will continue protecting against blood-feeding *Anopheles* even if they are damaged or torn(31) (page 28, line 387-390).**

CONCLUSION:

There is no evidence provided in this study about whether BCC might actually affect net lifetimes. Suggest dropping this statement entirely.

**Our study was not investigating the utility of BCC- however, it does seem logical to assume that if users realised that torn nets were still effective, despite having holes in them, that this might increase the lifespan of the nets. We have modified the sentence to say “****It is important that users are aware of the benefits of using nets, even if they become torn- and targeted behaviour change communication campaigns may help to reduce the attrition of torn nets.” (line 416-419)**

Neither of these statements can be concluded based on data and findings presented here. In fact don't you actually report that the shorter lived nets had a greater impact in this study? I recognize that this is confounded by the inclusion of PBO in those nets, but from this data and the way it was analyzed, it seems net life is actually inversely related to impact.

I'd suggest instead that you stick closer to the data, and assert that these are not meeting a three year median lifetime in this setting. ITs not clear at all what making a longer lasting net will do to overall impact from the data and analysis you present here. Additionally increasing campaign frequency to make up for net lifetime has not been analyzed here, so i would refrain from drawing conclusions about it. This would result in a dramatic increase in cost, it may itself have the effect of shortening net life times. I think its fine to clearly state that the additional advantage of PBO nets relative to regular nets seem to be less meaningful or not existent after two years, which suggest the possibility of greater impact with a shorter campaign cycle, but to make that recommendation without actually analyzing it seems a step too far.

**Our data shows that prevalence increases at the nets age in both arms, it therefore seems logical to suppose that if the nets lasted longer then the prevalence would be lower for longer. Additionally, it is clear from wider evidence (entomological studies etc) that the reduced prevalence in the PBO arm is not due to shorter lifespan of the nets (which would also be illogical) but to the PBO.**

**Whilst we have not analysed the impact of increasing campaign frequency, that does not preclude us discussing it in the discussion of a paper related to the durability of nets. We have discussed our results in light of other findings and observed that none of the net life span meet the WHO criteria of 3 years. This final statement is not an overinterpretation of our data but a recommendation following the evidence generated by our study in light of other bodies of evidence.**

**We have reworded the statement slightly so it now reads "Moving forward, to ensure population coverage with effective nets, either manufacturers need to make nets that last for the recommended 3 years, in terms of fabric and chemical content, or distribution methods need to be increased in regularity to ensure that the population is able to sleep under effective bed nets.” (line 419-422)**

Reviewer #2: This paper answers an important question and was clearly written.

I have only minor comments:

Methods:

I would rename the section called "Study area" since it contains information on the arms of the trial this study is embedded in, not just the study area.

**Thank you for the suggestion. The section has been renamed and now reads as “Study area and study arms” (page 4, line 81)**

Table 1: The authors could add a column on how many clusters each component was measured in.

**This has been completed (Table 1) (line 147)**

Table 1: Is survivorship just 1 - attrition? if so there's no need for both. If not, a better explanation of the difference should be given.

**We thank the reviewer for this interesting point. Yes, survivorship is 1-attrition. Only attrition has been kept in the tables and survivorship been taken out.**

The section on the "longitudinal LLIN study" has information about the cross-sectional study, which is confusing because there is a separate section on this. It would be easier to keep the information on each study in its own section.

**This has now been corrected. The sentence that had information about the cross-sectional study has been moved to the next section under the cross-sectional study section (page 5, line 111-113).**

In the section on "cross-sectional LLIN study" could the authors specify the time points so avoid confusion with the longitudinal study.

**The sentence has been moved here from the longitudinal study paragraph and now the first sentence to this paragraph on page 5 reads “Net durability was also assessed in 480 households (10 households per cluster) during successive cross-sectional surveys done at 4, 9, 16, 22, 28, and 33 months post-distribution (Table 1)” (page 6, line 124-125).**

Results:

Table 3: The 3 attrition different rates should be explained in table 1.

**Thank you for the useful comment. We have now included a description of different attrition rates in (Table 1) (line 147)**

table 3: the "attrition rates" are not actually rates (rates have a denominator of time) so please just say attrition.

**We agree that attrition rate is probably not the best to describe the outcomes however this is the word that is used in WHO guidelines for monitoring durability of nets in field condition and commonly used by other authors. We would like to keep it for consistency with other related publications**. (<https://apps.who.int/iris/handle/10665/44610>)

Kind Regards,

Eliud Lukole
